# Hip Positioning and Sitting Posture Recognition Based on Human Sitting Pressure Image

**DOI:** 10.3390/s21020426

**Published:** 2021-01-09

**Authors:** Qilong Wan, Haiming Zhao, Jie Li, Peng Xu

**Affiliations:** 1 College of Mechanical and Electrical Engineering, Central South University, Changsha 410083, China; wanqilong@csu.edu.cn (Q.W.); jieli_me@csu.edu.cn (J.L.); 193712116@csu.edu.cn (P.X.); 2 State Key Laboratory of High Performance Complex Manufacturing, Central South University, Changsha 410083, China

**Keywords:** sitting pressure image acquisition system, hip positioning algorithm, support vector machine, sitting posture classification

## Abstract

Bad sitting posture is harmful to human health. Intelligent sitting posture recognition algorithm can remind people to correct their sitting posture. In this paper, a sitting pressure image acquisition system was designed. With the system, we innovatively proposed a hip positioning algorithm based on hip templates. The average deviation of the algorithm for hip positioning is 1.306 pixels (the equivalent distance is 1.50 cm), and the proportion of the maximum positioning deviation less than three pixels is 94.1%. Statistics show that the algorithm works relatively well for different subjects. At the same time, the algorithm can not only effectively locate the hip position with a small rotation angle (0°–15°), but also has certain adaptability to the sitting posture with a medium rotation angle (15°–30°) or a large rotation angle (30°–45°). Using the hip positioning algorithm, the regional pressure values of the left hip, right hip and caudal vertebrae are effectively extracted as the features, and support vector machine (SVM) with polynomial kernel is used to classify the four types of sitting postures, with a classification accuracy of up to 89.6%.

## 1. Introduction

As society develops, more people work in office chairs. There are some health risks associated with this way of working, such as lumbar diseases [1], which are easy to cause because people tend to neglect their sitting posture when they focus on their work. Therefore, an algorithm which can intelligently recognize and provide feedback on human sitting posture is increasingly valuable.

At present, there are three main ways of sitting posture recognition, which are based on machine vision [2,3,4,5], wearable motion sensors [6,7,8,9] and external pressure sensors [10,11,12,13,14,15,16,17,18,19,20,21,22,23,24,25]. Although machine vision technology has achieved great success in the field of posture recognition [26], it is difficult to work normally in situations with many obstacles. For wearable motion sensors, a prominent problem is that they need to be worn by users, which is very inconvenient for many people. In contrast, external pressure sensors do not have the problems mentioned above. They can collect signals from the human body simply by being mounted on a chair. Therefore, it can be inferred that sitting posture recognition technology based on external pressure sensors has greater application prospects.

Many researchers have studied sitting posture recognition using external pressure sensors. Ma et al. [11] took the pressure values of twelve pressure sensors as features and used J48 algorithm to classify five types of sitting postures, obtaining 99.47% experimental classification accuracy. Roh et al. [12] took the pressure values of four force measuring units as features and used support vector machine (SVM) to classify six types of sitting posture, obtaining 97.20% experimental classification accuracy. Ahmad et al. [13] used a 4 × 4 matrix pressure sensor to classify four types of sitting postures by decision tree algorithm, obtaining about 80% experimental classification accuracy. Kim et al. [24] used an 8 × 8 matrix pressure sensor and convolutional neural network algorithm to classify five types of sitting postures, obtaining 95.3% experimental classification accuracy. The above sitting posture recognition algorithms did not predict the position of the hip and extracted the pressure features according to the position of the hip, but directly selected the pressure value of the fixed position of the seat as features for sitting posture recognition. This approach has certain risks, because the obtained prediction models are likely to perform well only for sitting postures of specific angles and positions, but not for other angles and positions. The major contributions of this paper are as follows:

- A sitting pressure image acquisition system is designed, which can conveniently and effectively collect the human sitting pressure image.
- An innovative hip positioning algorithm based on hip templates is proposed. The algorithm can effectively locate the hip position by using the human sitting pressure image and can adapt to the sitting posture with different angles.
- Based on the above hip positioning technology, this paper effectively extracts the pressure values of the left hip area, right hip area and caudal area as features. It also explores the optimal parameters of SVM with polynomial kernel, Gaussian kernel and Sigmoid kernel for the classification of four types of sitting postures.

## 2. Materials and Methods

### 2.1. System

**Figure 1:**
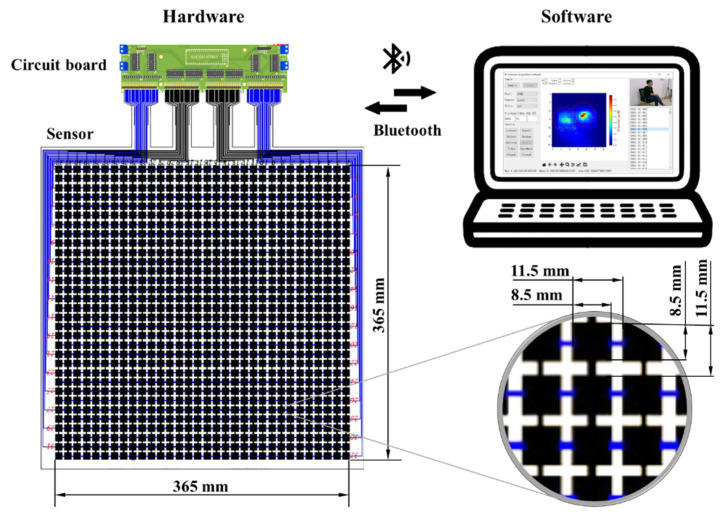
The sitting pressure image acquisition system.

The sitting pressure image acquisition system is shown in Figure 1, which mainly includes two parts: hardware and software.

Hardware includes sensor and circuit board. The sensor is a 32 × 32 pressure sensor array with a sensing area of 365 × 365 mm. It has a total of 1024 sensing units, each of which has a size of 8.5 × 8.5 mm and a pressure measurement range of 0–100 N. The distance between adjacent sensing units is 11.5 mm, which means that the distance of one pixel on the pressure image is equivalent to 11.5 mm. The circuit board takes the STM32F103C8T6 as the control chip and uses the 74HC595 and 4051 chips to expand the IO (input and output ports) and analog channels of the control chip to complete the point-by-point scan acquisition of the pressure sensor array. The circuit board and the computer communicate via Bluetooth. The price of the hardware is very low, no more than $150. Table 1 shows the names, prices and quantities of components included in a set of hardware. In contrast, the commercially available Body Pressure Measurement System (BPMS) developed by TekScan [27] costs more than $10,000. In addition, the cost of the hardware is lower than that of Ma et al. [11].

**Figure 2:**
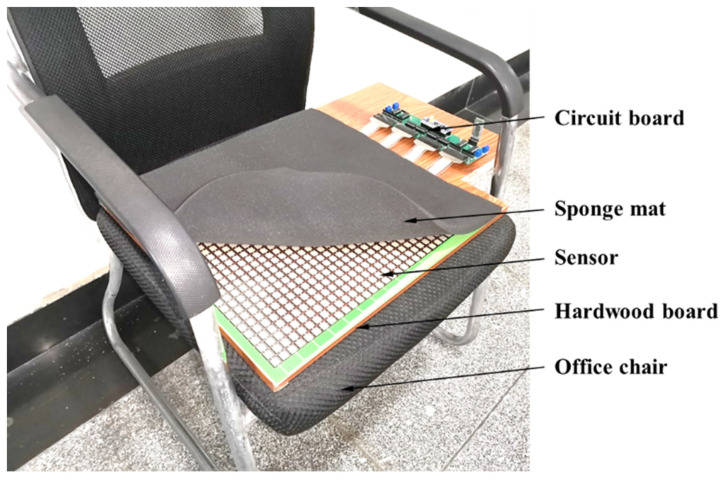
Hardware layout.

The hardware is laid on an office chair with armrests and a backrest. The bottom of the sensor is a smooth hardwood board, and the surface of the sensor is covered with a 1-cm-thick sponge mat, as shown in Figure 2.

The software is written in Python, which can efficiently collect, store, manage and present pressure images. Moreover, it also has the function of taking photos and marking hip position, which can greatly facilitate the acquisition, marking and storage of pressure images.

### 2.2. Data Acquisition and Marking

This study involved ten healthy adult subjects, including nine males and one female. The statistical information of their age, weight and height has been presented in Table 2. It can be found that the height and weight of the ten subjects are quite different, ranging from 160 cm to 180 cm and 51 kg to 84 kg. Although there is gender imbalance in these subjects, this has no effect on this study, because there is no obvious difference in sitting pressure images between males and females with the same height and weight.

This study aims to identify four types of sitting postures, including sitting upright, leaning back, leaning left and leaning right. To simulate people’s complex sitting postures, three variations are set for each type of sitting posture, namely, normal sitting, left leg crossed and right leg crossed, as shown in Table 3.

During data acquisition, the subjects were asked to sit upright, lean back, lean left and lean right on the experimental seat. Each person collected 20 pressure images for each type of sitting position, including five with normal sitting, five with left leg crossed and five with right leg crossed. For the remaining five pressure images, subjects could pose at will on the premise of ensuring the basic requirements. Therefore, a total of 800 pressure images were collected in this experiment. In this process, each time a pressure image was collected, the subject was asked to rotate a certain angle and change the position of the hip, but the position of their hands was not limited. The positions of the left and right hips were immediately manually marked and stored for subsequent comparison with the hip positions predicted by the algorithm.

### 2.3. Data Preprocessing

**Figure 3:**
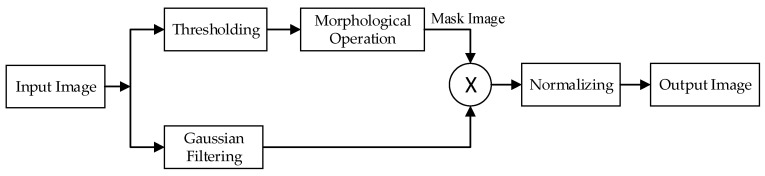
Preprocessing flow chart of pressure images.

Due to external interference, there may be noise in the pressure images. To improve the quality of the images, unified image processing is needed. As shown in Figure 3, the process is divided into two paths. The path above represents the conversion of a pressure image into a binary mask image using a threshold (its value is 0.45 times the average value of the pressure image), followed by morphological operations (open operation first, then closed operation) to improve the mask quality. The path below represents Gaussian filtering (filter size is 3 × 3) for the pressure image. Then, the images obtained by the two paths are multiplied to obtain the image after noise reduction. Finally, to eliminate the influence of the weight difference of the subjects, L1 normalization is performed on the image. Its equation is

(1) $$dst\left(i,j\right)=\frac{scr\left(i,j\right)}{\sum scr\left(x,y\right)},$$

where scr(i,j) and dst(i,j) respectively represent the pixel value of the ith row and jth column of the image before and after normalization; ∑scr(x,y) represents the sum of the values of all pixels in the image before normalization.

### 2.4. Hip Positioning Algorithm Based on Hip Templates

#### 2.4.1. Principle of the Algorithm

**Figure 4:**
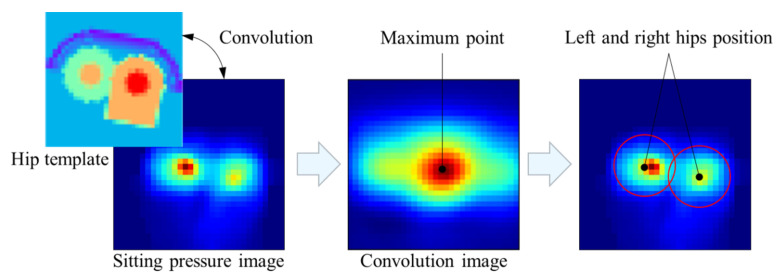
The basic process of the hip positioning algorithm.

As shown in the Figure 4, the basic process of the hip positioning algorithm is as follows:

1. Using the hip template to convolve the sitting pressure image to get the convolution image.
2. Finding the position of the maximum point in the convolution image, which will be considered as the best position.
3. Calculating the position of the left and right hips from the position of the maximum point and the size of the template.

The key to the algorithm is to make the convolution value of the template as large as possible in the actual position of the hip and as small as possible in other positions. Therefore, the hip template needs to cater to the hip pressure image as much as possible.

#### 2.4.2. The Design of Hip Templates

**Figure 5:**
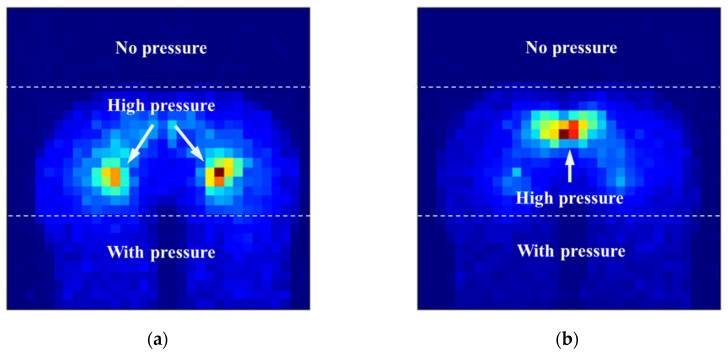
Typical sitting posture pressure images. (**a**) The central area of the left and right hips is a high-pressure area. (**b**) The area of the caudal vertebra is a high-pressure area.

Via the observation of a large number of sitting pressure images, it can be found that the sitting pressure images have the following characteristics, as shown in Figure 5:

- The central area of the left and right hips and the area of the caudal vertebra are often high-pressure areas; and
- There is pressure in the front of the hips, and no pressure in the rear of the hips.

**Figure 6:**
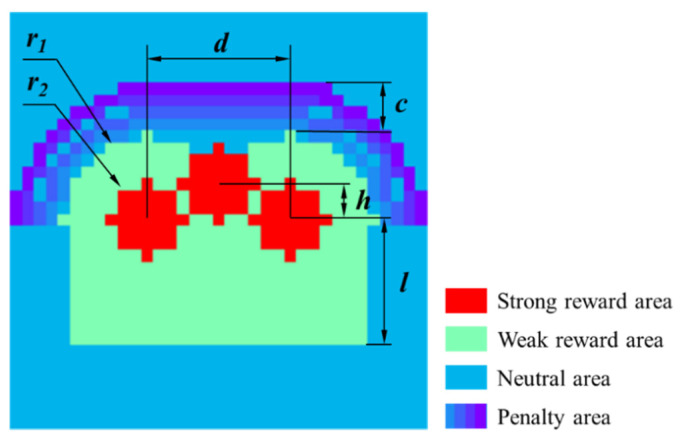
Hip template.

According to above characteristics, we specially designed a hip template, as shown in Figure 6. The hip template is divided into three parts: reward area, punishment area and neutral area. The value of the reward area is positive, and the value of the strong reward area is larger than that of the weak reward area. The strong reward area is set in the center area of the left and right hips and the area of the caudal vertebra to cater to possible high-pressure characteristics. The penalty area is located above the hips and is in the shape of half a runway. The value of this area is negative, and the farther away from the inner circle, the smaller the value. The neutral area is the part excluding the reward area and punishment area, and its value is zero. With reference to the hip size of the average adult, we set the radius of the left and right hips $r_{1}$ to seven pixels, the radius of the three strong reward areas $r_{2}$ to three pixels, the distance between the center of the left and right hips d to twelve pixels, the vertical distance from the center of the caudal vertebra to the center of the two hips h to three pixels, the length of the rectangle extending in front l to nine pixels and the thickness of the penalty area c to four pixels, as shown in Table 4.

**Figure 7:**
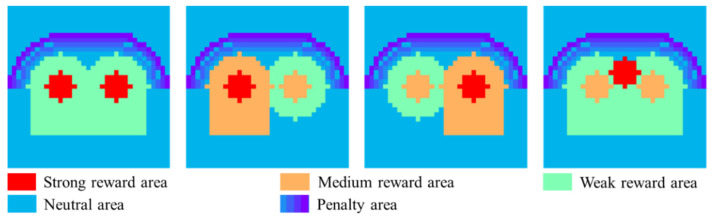
Improved four hip templates.

Since the high-pressure areas at the left hip, right hip and caudal vertebra generally do not appear at the same time, the template in Figure 6 does not cater well to the human sitting pressure images. Therefore, based on the dimensions and shape of Figure 6, we designed four hip templates to suit possible situations, as shown in Figure 7. The red area, orange area and cyan area are respectively denoted as strong reward area, medium reward area and weak reward area. Their values are $v_{1}$, $v_{2}$ and $v_{3}$ ($v_{1}>v_{2}>v_{3}$). The blue area is the neutral area, and the value is zero. The purple gradient area is the penalty area. The farther from the hip area, its value decreases linearly (starting from zero and the linear decrease coefficient is k).

To make the positioning effect better, several techniques are used here:

- With reference to the first template, the pixel values of the reward areas of the remaining three templates are scaled equally so that the sum of the pixel values of the reward areas of the four templates are the same. The purpose is to ensure that each template has the same weight in the process of convolution.
- To locate the hip position of the sitting posture with different rotation angles, the four templates are rotated in the same way. Since it is almost impossible for a person to sit on an office chair with armrests with a rotation angle greater than 40°, the rotation range is set from −40° to 40°. The specific rotation angles include −40°, −30°, −20°, −10°, 0°, 10°, 20°, 30° and 40°. Therefore, the final number of templates is 36.

#### 2.4.3. The Process of Hip Positioning

Hard objects on the hip, such as the hip bone, will cause too steep pressure peaks in the sitting pressure image, which is not good for the hip positioning algorithm. Therefore, all pressure images are uniformly weakened. The equation is

(2) dst(i,j)=min(scr(i,j),MeanVal×WeakenCoef),

where scr(i,j) and dst(i,j) respectively represent the value of the point in the ith row and jth column of the image before and after processing; MeanVal represents the pressure average value of the pressure area of the pressure image; WeakenCoef represents the weakening coefficient, which is an adjustable constant.

The procedure of the hip positioning based on the hip templates is as follows:

1. Using Equation (2) to weaken the pressure image.
2. The pressure image is convolved with the 36 templates respectively to obtain 36 convolution images.
3. Finding the position of the maximum value and the angle of the corresponding template in 36 convolution images and taking them as the best predicted position and angle.
4. Calculating the position of the left hip and the right hip via the best predicted position and angle.

#### 2.4.4. Parameters Optimization

The choice of template parameters affects the final performance of the algorithm. The parameters that have not been determined include the weak reward area value $v_{1}$, the medium reward area value $v_{2}$, the strong reward area $v_{3}$, the linear decrease coefficient k and the weakening coefficient WeakenCoef. It is meaningless if all parameters are scaled in the same proportion, so we directly set $v_{1}$ to one as the reference value. Therefore, only four parameters need to be optimized.

The closer the predicted position of the hips to the manually marked position, the better the algorithm performance. Therefore, the average deviation between the predicted position of the hips and the manually marked position is taken as the optimization goal, namely

(3) $$\min\frac{\sum_{i=1}^{N}\left(dist_{li}+dist_{ri}\right)}{2\times N},$$

where N represents the number of all pressure images; $dist_{li}$ and $dist_{ri}$ respectively represent the positioning deviation of the left hip and right hip of the ith pressure image.

Since the objective function is not continuous with respect to parameters, the conventional gradient descent method cannot be used for the optimization. Therefore, we adopted the grid search method to find the approximate optimal solution.

### 2.5. Sitting Posture Recognition

#### 2.5.1. Features Selection and Processing

**Figure 8:**
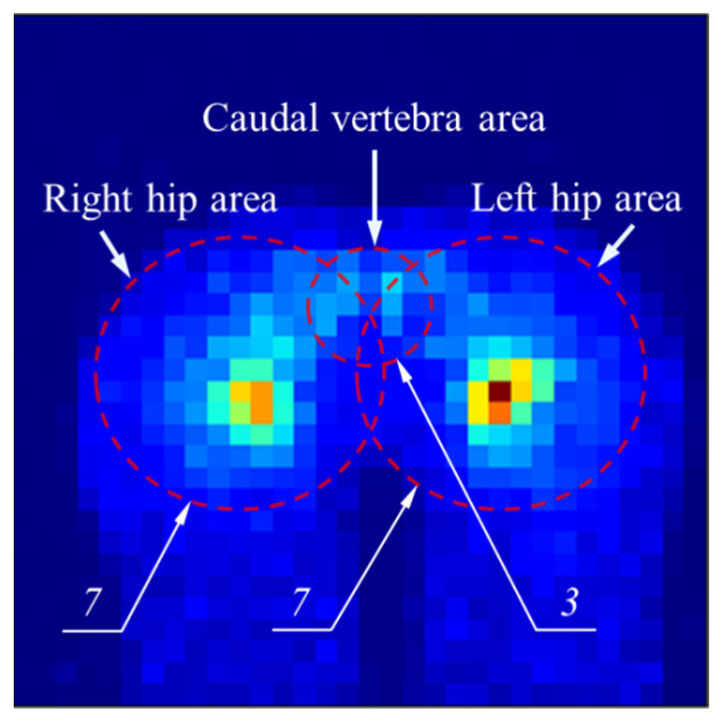
Schematic diagram of left hip area, right hip area and caudal vertebra area.

After completing the hip positioning of the pressure image, the center positions of the left hip, right hip and caudal vertebra can be calculated according to the template size. In this study, three features were extracted, which were the sum of the regional pressure values of the left hip, right hip and caudal vertebrae. The shape of the three areas is round, and the radius is set as seven pixels, seven pixels and three pixels respectively, as shown in Figure 8. To avoid possible skewness of the data, the values of the three features are standardized. The equation is

(4) $${\tilde{x}}_{ij}=\frac{x_{ij}-{\bar{x}}_{i}}{\sigma_{i}},$$

where i represents the code number of the features; j represents the code number of the pressure images; $x_{ij}$ and ${\tilde{x}}_{ij}$ represent the value of the ith feature of the jth pressure image before and after standardization; ${\bar{x}}_{i}$ represents the average value of the ith feature; $\sigma_{i}$ represents the standard deviation of the ith feature.

#### 2.5.2. Classification

**Figure 9:**
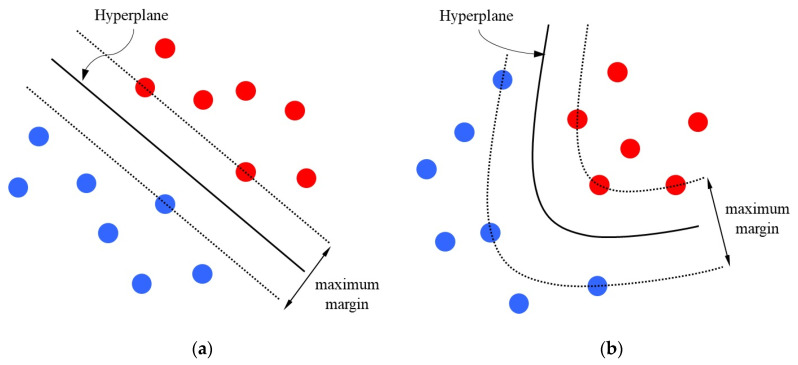
Schematic diagram of SVM. (**a**) Ordinary SVM. (**b**) SVM with nonlinear kernel.

Support vector machine (SVM) is a widely used classifier with excellent generalization performance [28]. It can find a hyperplane with the maximum margin between two different types of data sets, as shown in Figure 9a. The general form of the hyperplane is

(5) $$\omega^{T}x+b=0,$$

where x, ω and b represent the input vector, the vector perpendicular to the hyperplane and the constant, respectively. If $y\left(x\right)=\omega^{T}x+b$, then the classification result of SVM can be expressed as

(6) $$\{\begin{array}{c} y\left(x\right)>0\to class1\\ y\left(x\right)<0\to class2 \end{array},$$

Since the hyperplane of a normal SVM is linear, it is difficult to classify the data in Figure 9b. To solve the problem, it is often necessary to add a nonlinear kernel inside SVM to improve its classification performance. As shown in Figure 9b, SVM with nonlinear kernel can better classify complex data. The general form of hyperplane of SVM with nonlinear kernel is

(7) $$\omega^{T}\varphi \left(x\right)+b=0,$$

where φ(x) represents the mapping of the input vector x in a higher-dimensional nonlinear space. Generally, $k\left(x_{i},x_{j}\right)=\varphi {\left(x_{i}\right)}^{T}\varphi \left(x_{j}\right)$, and $k\left(x_{i},x_{j}\right)$ is called the kernel function, where $x_{i}$ and $x_{j}$ represent two different input vectors.

In this study, SVM with polynomial kernel, Gaussian kernel and Sigmoid kernel were used to complete the classification task, and the grid search method was used to find the optimal parameters that could achieve the highest classification accuracy. Table 5 shows the parameters of SVM with different kernels. *C* represents the regularization coefficient, which is mainly used to improve the generalization performance of SVM.

Since SVM is a binary classifier, it needs to apply additional strategies for multi-classification tasks. This study adopted a one-to-all strategy [29] to solve the problem. This strategy takes one class as a positive example at a time and uses all the remaining classes as negative examples to train multiple binary classifiers to complete multi-classification tasks.

In the process of training and prediction, if the data is split between the training set and the test set only once, the final evaluation results will have a lot of randomness. To make the final evaluation result more stable and credible, we used five-fold cross validation to evaluate the model.

## 3. Results and Discussion

### 3.1. The Effect of Hip Positioning

**Figure 10:**
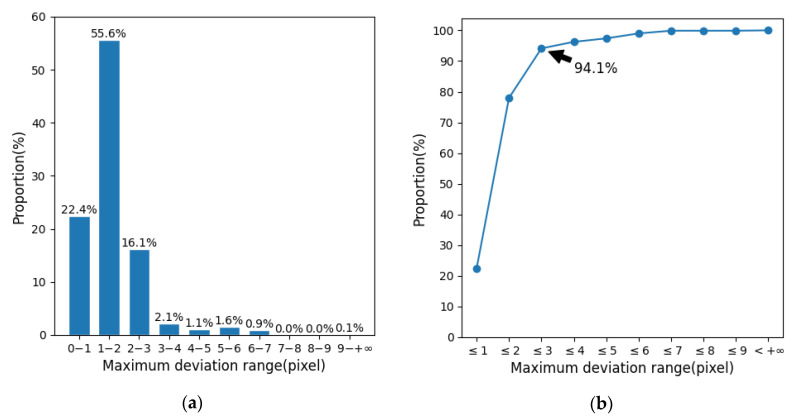
Statistics chart of the maximum positioning deviation of the images. (**a**) Statistics of the maximum positioning deviation of the images in each interval. (**b**) Cumulative curve of the distribution of maximum positioning deviation of the images.

Table 6 shows the optimization results of the template parameters. Using these parameters for hip positioning, the average deviation between the hip position predicted by the algorithm and the manually marked position is 1.306 pixels (the actual equivalent distance is 1.50 cm). We call the maximum value of the prediction deviation of the left and right hips of the image as the maximum positioning deviation of the image. Figure 10a shows the statistics of the maximum positioning deviation of the images in each interval. It can be found that the maximum positioning deviation of most images falls within the range of 0–1, 1–2 and 2–3 pixels, and the ratio of the range of 1–2 pixels is more than half reaching 55.6%. Figure 10b is the cumulative curve of Figure 10a. It can be intuitively seen that the accumulative value of the first three intervals has reached 94.1%, which indicates that most positioning deviations are small ones, no more than three pixels. This shows that the overall performance of the hip positioning algorithm in this paper is relatively good.

**Figure 11:**
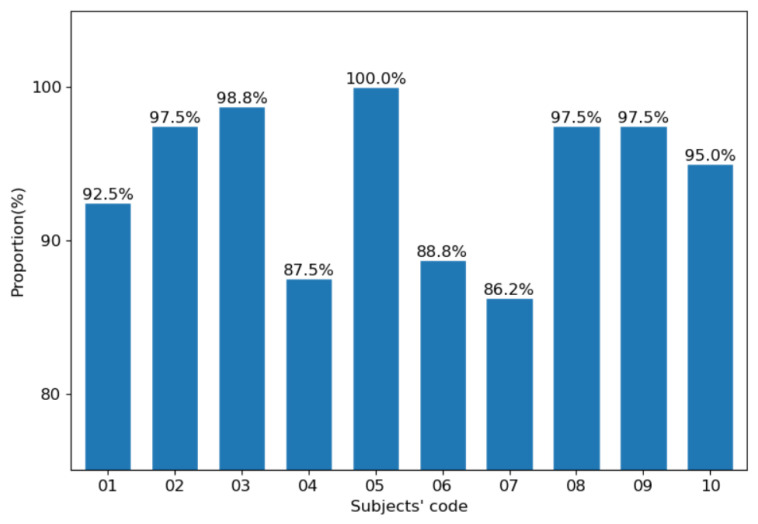
The proportion of the maximum positioning deviation of each subject less than three pixels.

Figure 11 shows the statistics of the proportion of the maximum positioning deviation of each subject less than three pixels. Seven out of ten are more than 92%. The highest proportion reached 100% and the lowest reached 86.2%. This shows that the hip positioning algorithm works relatively well for different subjects, although there are still differences.

**Figure 12:**
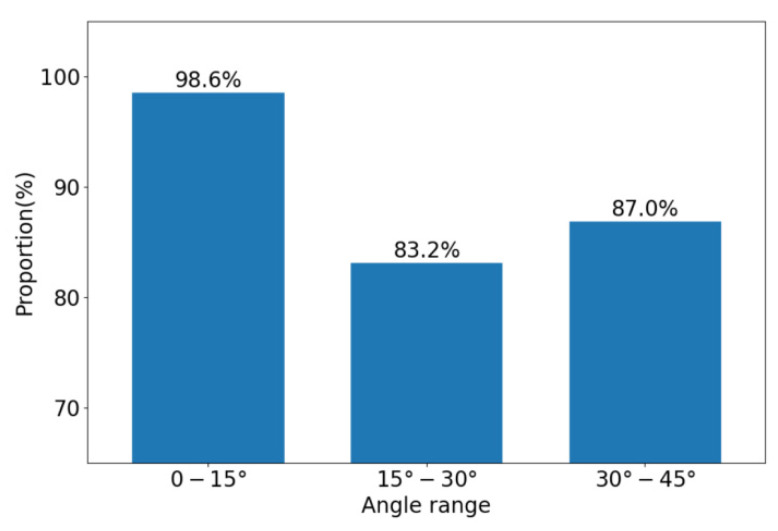
The proportion of the maximum positioning deviation less than three pixels in the three angle ranges.

Figure 12 shows the proportion of the maximum positioning deviation less than three pixels in the three angle ranges. It is worth noting that for the sitting posture with a small rotation angle (0°–15°), the proportion of the maximum positioning deviation less than three pixels is as high as 98.6%. For sitting postures with a medium rotation angle (15°–30°) and a large rotation angle (30°–45°), the proportion also reaches 83.2% and 87.0%, respectively. This shows that the hip positioning algorithm has high accuracy for the sitting posture with a small rotation angle (0°–15°), and it has certain adaptability to the sitting posture with a medium rotation angle or a large rotation angle.

### 3.2. The Effect of Sitting Posture Recognition

Table 7 shows the optimal parameters and best accuracy of SVM with different kernels. By comparison, it is found that the performance of SVM with polynomial kernel is the best, and the best accuracy rate is 89.6%.

**Figure 13:**
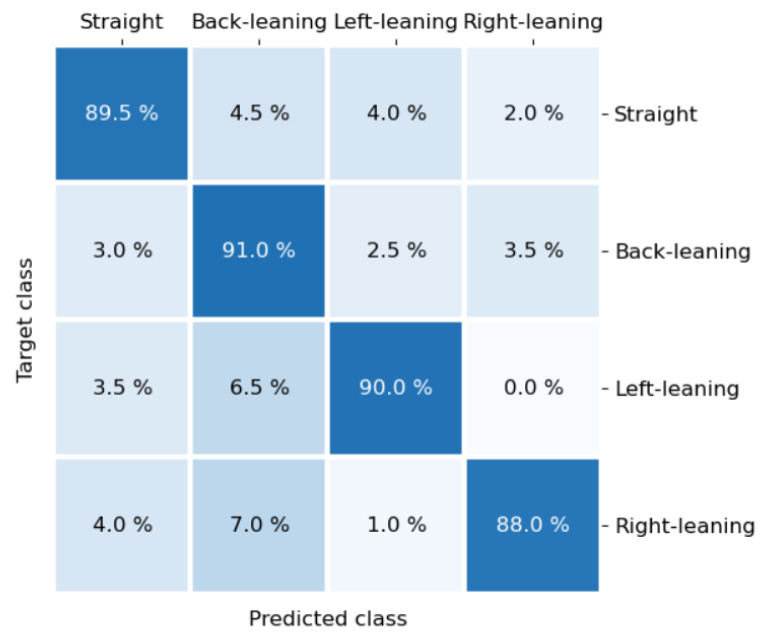
The confusion matrix classified by SVM with polynomial kernel.

Figure 13 is the confusion matrix classified by SVM with polynomial kernel. The classification accuracy and mutual misclassification of each sitting posture can be observed in detail in the confusion matrix. The accuracy of the four types of sitting postures is very close to the average accuracy (89.6%), which shows that the performance of the classification algorithm for the four types of sitting postures is relatively average. The two types of sitting postures, leaning left and leaning right, are the most distinct, and the total confusion between them is only 1.0%.

### 3.3. Comparison and Analysis

Table 8 shows the comparison between our study and previous studies. Our sitting posture recognition algorithm does not have obvious advantages in terms of the number of posture types and accuracy. However, it should be noted that this study asked subjects to change the angle and position of their hips in the process of data acquisition and deliberately set variations for each type of sitting posture, which greatly improves the difficulty of recognition. In addition, we only extracted three features, which is less than most other algorithms, but achieves 89.6% accuracy for the classification of four types of posture. This shows that our algorithm has great potential. We are also studying the extraction of more features, such as the pressure in the anterior hip area, to further improve the number of posture types and accuracy of classification.

For the hip positioning algorithm, its significance is not only limited to the feature extraction in this paper, but also provides new ideas for the performance improvement of other algorithms, such as deep learning. The algorithm can be used to correct sitting pressure images with rotation, and the corrected images are obviously more easily distinguished by neural networks or other algorithms than images with rotation. In addition, in some cases, the prediction of hip position is more important than the prediction of sitting posture, such as the prevention of hip pressure ulcers in people who have difficulty with movement. This field is more concerned with the pressure time of each position of the hip. If the hip position can be located, the pressure time of each position of the hip will be easy to calculate. Therefore, the hip positioning algorithm has practical significance.

Although the objects studied in this paper are all static pressure images and do not involve dynamic images, it is known that the basic unit of dynamic images is static images. Our research will contribute to the study of action prediction on the seat, which is of practical significance to ensure the safety of drivers [30].

Since all the data used in this paper are from healthy adults, our optimal parameters are only applicable to healthy young adults. For people with abnormal body shape or an incomplete body, we would need to collect the relevant pressure images and re-optimize the template parameters.

## 4. Conclusions

In this paper, a sitting pressure image acquisition system was designed. The system has low cost and can efficiently collect sitting pressure images. With the system, we innovatively proposed a hip positioning algorithm based on hip templates. The average deviation of the algorithm for hip positioning is 1.306 pixels (the equivalent distance is 1.50 cm), and the proportion of the maximum positioning deviation less than three pixels is 94.1%. Statistics show that the algorithm works relatively well for different subjects. At the same time, the algorithm can not only effectively locate the hip position with a small rotation angle (0°–15°), but also has certain adaptability to the sitting posture with a medium rotation angle (15°–30°) or a large rotation angle (30°–45°). Using the hip positioning algorithm, the regional pressure values of the left hip, right hip and caudal vertebrae are effectively extracted as the features, and support vector machine (SVM) with polynomial kernel is used to classify the four types of sitting posture, whose classification accuracy can be up to 89.6%.

In future work, we will extract more features of specific parts of the human body based on our hip positioning algorithm to further improve the accuracy of sitting posture classification and identify more sitting postures.

## Figures and Tables

**Table 1:** The names, prices and quantities of components included in a set of hardware.

Name	Unit Price (USD)	Quantity	Sum (USD)
The pressure sensor array	123	1	123
STM32F103C8T6	2	1	2
74HC595	0.3	4	1.2
4051	0.3	4	1.2
Bluetooth (HC-05)	3	2	6
PCB	5	1	5
Others			<10
Total			<150

**Table 2:** The statistical information of subjects’ age, weight and height.

	Minimum	Maximum	Average	Standard Deviation
Age (years old)	21	25	23.4	1.65
Height (cm)	160	181	172.6	5.97
Weight (kg)	51	84	69.0	9.17

**Table 3:** Four types of sitting posture and corresponding three forms of change.

	Normal Sitting	Left Leg Crossed	Right Leg Crossed
Sitting upright	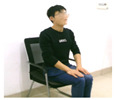	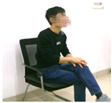	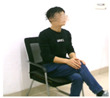
Leaning back	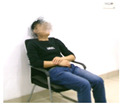	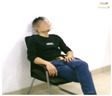	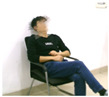
Leaning left	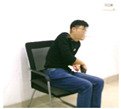	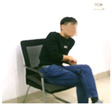	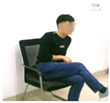
Leaning right	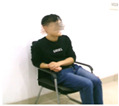	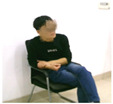	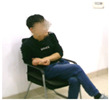

**Table 4:** Template size.

	*r*_1_	*r*_2_	*d*	*c*	*h*	*l*
Value (pixel)	7	3	12	4	3	9

**Table 5:** Parameter list of SVM with different kernels.

Kernel	Expression	Parameter
polynomial kernel	$k\left(x_{i},x_{j}\right)={\left(\beta x_{i}x_{j}+\theta \right)}^{d}$	C, β, θ, d
Gaussian kernel	$k\left(x_{i},x_{j}\right)=\exp\left(-\gamma \|x_{i}-x_{j}{\|}^{2}\right)$	C, γ
Sigmoid kernel	$k\left(x_{i},x_{j}\right)=\tanh\left(\beta x_{i}x_{j}+\theta \right)$	C, β, θ

**Table 6:** Optimization result of template parameters.

	*v*_1_	*v*_2_	*v*_3_	*k*	*WeakenCoef*
Value (pixel)	1	2	5	−0.3	2.8

**Table 7:** The optimal parameters and best accuracy of SVM with different kernels.

Kernel	Optimal Parameters	Best Accuracy
polynomial kernel	C=1.3, β=1.4,θ=0.8,d=3	89.6%
Gaussian kernel	C=0.2,γ=2	89.5%
Sigmoid kernel	C=0.4, β=0.2, θ=0.2	87.0%

**Table 8:** The comparison between our study and previous studies.

Author	Ask Subjects to Change the Angle and Position of Their Hips	Set Variations for Each Type of Posture	Number of Features	Number of Posture Types	Accuracy
Ma et al. [11]	No	No	12	5	99.47%
Roh et al. [12]	No	No	3	6	97.2%
Kim et al. [18]	No	No	64	5	95.3%
Zemp et al. [23]	No	No	17	7	90.9%
Xu et al. [21]	No	No	Unknown	7	85.9%
Meyer et al. [20]	No	No	1046	16	82%
Ahmad et al. [14]	No	No	19	4	80%
Our study	Yes	Yes	3	4	89.6%

## Data Availability

The data presented in this study are available on request from the corresponding author. The data are not publicly available due to the data involves personal privacy.
